# NIR-Based Electronic Platform for Glucose Monitoring for the Prevention and Control of Diabetes Mellitus

**DOI:** 10.3390/s24134190

**Published:** 2024-06-27

**Authors:** William Oñate, Edwin Ramos-Zurita, Juan-Pablo Pallo, Santiago Manzano, Paulina Ayala, Marcelo V. Garcia

**Affiliations:** 1 Carrera de Electrónica y Automatización, Universidad Politecnica Salesiana (UPS), Quito 170146, Ecuador; wonate@ups.edu.ec; 2 Faculty of Systems, Electronics and Industrial Engineering, Universidad Tecnica de Ambato (UTA), Ambato 180206, Ecuador; eramos7283@uta.edu.ec (E.R.-Z.); juanppallo@uta.edu.ec (J.-P.P.); victorsmanzano@uta.edu.ec (S.M.), ep.ayala@uta.edu.ec (P.A.); 3 Departamento de Ingeniería de Sistemas y Automática, University of the Basque Country, Euskal Herriko Unibertsitatea/Universidad del País Vasco, 48013 Bilbao, Spain

**Keywords:** non-invasive glucometer, near-infrared spectroscopy, NIR, non-invasive measurement

## Abstract

The glucose level in the blood is measured through invasive methods, causing discomfort in the patient, loss of sensitivity in the area where the sample is obtained, and healing problems. This article deals with the design, implementation, and evaluation of a device with an ESP-WROOM-32D microcontroller with the application of near-infrared photospectroscopy technology that uses a diode array that transmits between 830 nm and 940 nm to measure glucose levels in the blood. In addition, the system provides a webpage for the monitoring and control of diabetes mellitus for each patient; the webpage is hosted on a local Linux server with a MySQL database. The tests are conducted on 120 people with an age range of 35 to 85 years; each person undergoes two sample collections with the traditional method and two with the non-invasive method. The developed device complies with the ranges established by the American Diabetes Association: presenting a measurement error margin of close to 3% in relation to traditional blood glucose measurement devices. The purpose of the study is to design and evaluate a device that uses non-invasive technology to measure blood glucose levels. This involves constructing a non-invasive glucometer prototype that is then evaluated in a group of participants with diabetes.

## 1. Introduction

Diabetes mellitus is a chronic disease that affects millions worldwide and has significant implications for public health and healthcare systems. Regular monitoring of blood glucose levels is crucial for effective diabetes management and prevention of complications. Advances in sensor technology and electronic devices have paved the way for innovative and effective glucose monitoring systems [1].

Traditional methods for monitoring blood glucose levels involve invasive techniques such as finger-prick tests or continuous glucose monitoring systems that require subcutaneous sensor insertion [2,3]. These invasive approaches can cause pain, discomfort, and an increased risk of infection, potentially leading to poor patient compliance and suboptimal glycemic control [4].

Invasive glucometers, which are widely marketed globally, allow diabetes patients to monitor their glucose levels throughout the day without visiting a medical center [5]. However, these devices require frequent skin punctures or patch implants, leading to pain, infection risks, and additional expenses for lancets and test strips [6,7]. Minimally invasive devices, while reducing discomfort, still necessitate periodic replacement of skin-implanted patches.

Near-infrared spectroscopy (NIR) has emerged as a promising non-invasive alternative for glucose measurement, offering a less intrusive method compared to traditional techniques [8]. Despite the availability of various devices for glucose measurement, most remain invasive or semi-invasive, posing challenges for user adaptation due to their design and lack of portability.

NIR spectroscopy relies on the interaction between NIR light and biological tissues, wherein specific wavelengths are absorbed or scattered by glucose molecules, enabling quantitative analysis of glucose concentrations [9]. However, accurate and reliable measurement of blood glucose levels using NIR spectroscopy remains a significant challenge due to the complexity of biological tissues, interference from other substances, and the need for robust signal processing and calibration algorithms [10].

Various principles and methods for glucose sensing have been explored in the literature, each with its unique advantages and limitations. Electrochemical glucose sensors, which rely on enzymes or non-enzymatic approaches, have been widely investigated. Enzyme-based electrochemical sensors, utilizing glucose oxidase or other enzymes, offer high sensitivity and selectivity but suffer from limited stability and biocompatibility issues [11]. Non-enzymatic electrochemical sensors, often based on nanomaterials or nanostructures, exhibit improved stability and resistance to interferents but may have lower sensitivity and require complex fabrication processes [12].

Optical glucose sensors, including fluorescence-based and surface plasmon resonance (SPR) techniques, have garnered interest due to their non-invasive nature and potential for continuous monitoring [13]. Fluorescence-based sensors exploit the interactions between glucose and fluorescent molecules or nanoparticles, offering high sensitivity, but they face challenges in terms of specificity and biocompatibility [14]. SPR-based sensors detect changes in the refractive index caused by glucose binding, providing label-free and real-time monitoring capabilities but requiring complex instrumentation and surface functionalization [15].

Affinity-based glucose sensors, such as boronic-acid-based sensors and glucose-binding-protein-based sensors, have also been explored [16,17]. These sensors rely on reversible binding interactions between glucose and specific receptors, offering high selectivity but often suffering from limited sensitivity and potential interference from other biomolecules [18,19].

This pioneering work holds significant importance in the field of diabetes management and non-invasive biosignal monitoring. The highlights of the article are:

- This work presents a groundbreaking electronic biosignal monitoring system that employs near-infrared spectroscopy for non-invasive measurement of blood glucose levels, addressing a critical challenge in diabetes management.
- The proposed system offers a pain-free and convenient alternative to traditional invasive glucose monitoring techniques, potentially improving patient compliance and quality of life for individuals with diabetes.
- Through rigorous evaluation and validation, this study demonstrates the accuracy and reliability of the developed system, paving the way for its potential integration into clinical practice and revolutionizing the approach to continuous glucose monitoring.

The manuscript is structured to provide a comprehensive overview of the development and validation of a non-invasive glucose monitoring system. Section 2 highlights the advantages and limitations of current technologies, setting the stage for the innovative approach presented in our research. Section 3 details the design and development of the prototype, including the integration of near-infrared (NIR) sensors, signal processing algorithms, and wireless communication technologies. Section 4 presents the performance evaluation of the prototype: comparing its accuracy and reliability against traditional invasive methods. The Discussion Section (Section 5) interprets the findings and highlights the advantages of the non-invasive system, addresses potential limitations, and suggests areas for future research. Finally, the Conclusion (Section 6) summarizes the key outcomes of the study, emphasizing the potential impact on diabetes management and future applications of the technology.

## 2. Literary Review and State of the Art

This section provides a comprehensive review of the existing literature on glucose monitoring technologies. We examine the evolution of glucose sensing methods, including invasive, minimally invasive, and non-invasive techniques. Special attention is given to the advancements in electrochemical sensors and their applications in diabetes management. We also explore the role of nanomaterials and nanostructures in enhancing sensor performance. Additionally, we review recent studies on near-infrared spectroscopy (NIR) and its potential for non-invasive glucose measurement.

### 2.1. Literary Review

A number of studies have explored the potential of near-infrared (NIR) spectroscopy for non-invasive glucose monitoring in diabetes. Sridevi [20] and Castro-Pimentel [21] both demonstrated the strong correlation between non-invasive device results and real glucose concentrations, with Dorsaf [22] achieving a correlation of 0.97, and 97.33% of measurements falling within clinically acceptable regions. Hina [23] and Joseph [24] further discussed the potential of NIR technology for non-invasive glucose monitoring, with Hina [23] focusing on NIR photoplethysmography for blood glucose prediction and Joseph [24] providing a comprehensive review of non-invasive glucose sensors, including NIR spectroscopy. These studies collectively highlight the promise of NIR spectroscopy for non-invasive glucose monitoring in diabetes.

Several research studies have explored non-invasive glucose monitoring systems. A study by Jeyapriya and Ramalaskshmi [25] proposed a continuous glucose monitoring system (CGMS) to provide real-time glucose readings, highlighting the limitations of existing glucose sensors that require finger pricks. Although minimally invasive and non-invasive CGM systems are available, their high cost and requirement for finger-prick calibrations pose challenges, particularly in low- and middle-income countries [23]. This review explores various non-invasive glucose measuring technologies, including optical, transdermal, and enzymatic methods, with a particular emphasis on near-infrared (NIR) technology and NIR photoplethysmography (PPG) for blood glucose prediction. The review highlights the process of feature extraction from PPG signals and the application of machine learning methods for glucose prediction, concluding with insights and key points for the future development of PPG-NIR-based glucose monitoring systems.

Electrochemical sensors, including non-enzymatic sensors based on nanomaterials or nanostructures, have also been widely used in glucose monitoring. These sensors offer advantages such as high sensitivity and selectivity, but they come with disadvantages like potential interference from other substances and the need for frequent calibration. Recent studies have explored the integration of wearable sensors and artificial intelligence for continuous health monitoring, as demonstrated by researchers from the University of California, San Diego (2021), and the Massachusetts Institute of Technology (2020) [26].

Despite these advancements, the challenge remains to develop a non-invasive, accurate, and user-friendly glucose monitoring system. This article presents the design and development of an electronic biosignal monitoring system using near-infrared (NIR) sensors for non-invasive glucose measurement. The system integrates signal processing and wireless communication technologies to provide continuous and accurate monitoring of glucose levels, heart rate, and oxygen saturation.

The results of the studies are encouraging: most have demonstrated accuracy and reliability in NIR glucose measurement. However, some studies have identified that accuracy can be affected by factors such as interference from other components in the blood and tissue, skin variability, and body temperature. Despite these challenges, non-invasive glucose measurement with NIR has the potential to revolutionize diabetes management. Advances in research, improved accuracy, reliability, and affordability of NIR technology make it a valuable tool for patients with diabetes and their caregivers in the near future. Near-infrared spectroscopy opens a hopeful path toward more effective, painless, and convenient glycemic control for people with diabetes, allowing them to improve their quality of life and prevent complications associated with the disease.

It is essential to highlight the importance of researching and developing non-invasive principles for the measurement of biomarkers such as glucose, as this not only improves the quality of life of patients but also facilitates continuous monitoring and informed clinical decision making. In this paper, we present in detail the main non-invasive methods for glucose measurement, analyze their advantages and limitations, and discuss the feasibility of their implementation in the commercial setting for the benefit of the medical community and patients [27].

### 2.2. Diabetes Mellitus

Diabetes mellitus, commonly known as diabetes, is a chronic disease that develops when the pancreas cannot produce enough insulin or when the body cannot effectively use the insulin it produces. Insulin is a crucial hormone that acts as a key and allows glucose from the foods we consume to enter the body’s cells for energy production. All carbohydrate-containing foods break down into glucose in the blood, and insulin facilitates its entry into cells.

The lack of insulin production or effective utilization leads to elevated blood glucose levels, known as hyperglycemia. This prolonged condition is associated with damage to various organs and tissues in the body. There are several types of diabetes, with the most common being type 2 diabetes, previously known as non-insulin-dependent diabetes or adult-onset diabetes, which accounts for at least 90% of cases. It is characterized by insulin resistance, a relative deficiency of insulin, or both. This type of diabetes can be diagnosed at any stage of life and often goes undetected for many years until complications arise or routine blood tests or urine glucose tests are performed.

Type 2 diabetes is often associated with being overweight or obese, which can cause insulin resistance and elevated blood glucose levels. Initially, individuals with type 2 diabetes can manage the condition through lifestyle changes such as exercise and diet. However, over time, most individuals require oral medications or insulin for treatment.

### 2.3. Traditional Glucose Measurement Methods

Measuring blood glucose levels is an essential part of managing diabetes. There are several traditional methods that have been used over time:

1. Blood glucose monitors (glucometers): These are devices that can measure the concentration of glucose in a blood sample. Typically, a small drop of blood, obtained by pricking a finger, is placed on a test strip that is inserted into the device. The glucometer then displays the blood glucose level at that moment. These devices are accurate, and the reading is obtained within seconds.
2. Hemoglobin A1c (HbA1c) test: This is a laboratory test that provides an overview of average blood glucose levels over the past 2 to 3 months. The test measures the percentage of hemoglobin, the protein in red blood cells that carries oxygen, that is coated with sugar (glycated). The target range for most people is less than 7%, but it may vary depending on each person’s situation.
3. Fasting blood glucose test: In this test, a person must fast for at least 8 h before a blood sample is taken. The results are used to assess how the body handles glucose after a period without food.
4. Oral glucose tolerance test (OGTT): This test measures the body’s response to glucose. It involves overnight fasting followed by the ingestion of a glucose solution. Blood samples are taken before and after glucose solution intake to measure how the body processes glucose.

All these methods have proven to be effective for diabetes management. However, with advanced technology, new methods of blood glucose monitoring are being developed and used, such as real-time glucose monitors and flash glucose monitoring systems, which can provide more frequent and detailed readings of glucose levels.

### 2.4. Non-Invasive Glucose Measurement Methods

Non-invasive glucose measurement methods are devices or technologies that can measure glucose levels without the need for skin pricking or blood extraction. These methods are an attractive alternative to traditional glucose monitoring methods, especially for those who need to check their glucose levels frequently [28,29,30]. Here are some of the non-invasive methods that have been developed:

1. Continuous glucose monitors (CGMs): Although some require a small needle to insert a sensor under the skin, there are advancements in developing completely non-invasive CGM sensors. These devices can provide nearly continuous glucose readings, and some can alert users if their glucose levels are too high or too low [31].
2. Flash glucose monitors: These devices, such as the FreeStyle Libre system, use a sensor attached to the body that measures glucose in the interstitial fluid (the fluid between cells). Although inserting the sensor requires skin pricking, once placed, readings can be taken simply by scanning the sensor with a reader or smartphone, eliminating the need for finger pricks for glucose measurements [32].
3. Spectroscopy technology: These non-invasive devices use light to analyze the chemical properties of body tissues and determine glucose levels. This can be done through the skin and does not require a blood sample [32].

### 2.5. Near-Infrared Spectroscopy

Near-infrared spectroscopy (NIR) is a non-destructive analytical method that utilizes the near-infrared region of the electromagnetic spectrum (approximately 780 nm to 2500 nm). It is based on molecular vibrations, with energy corresponding to the range of bond stretching and deformation vibrations in molecules [33].

In NIR spectroscopy, incident light is absorbed, reflected, or transmitted. The reflected or transmitted light can be analyzed to determine the chemical properties of the material being examined. This is because different molecules absorb light at different wavelengths.

NIR spectroscopy is a powerful tool that is widely used in various applications ranging from agriculture and the food industry to pharmaceuticals and medicine. In medicine, one of the applications being researched is the non-invasive measurement of blood glucose for individuals with diabetes.

The goal of using NIR spectroscopy to measure blood glucose is to obtain an accurate reading without the need for a skin puncture. In theory, NIR light can penetrate the skin and reflect back to the device, which then analyzes the reflected light to determine glucose levels. However, this is a significant technical challenge due to factors such as variation in skin composition among individuals and interference from other substances in the skin that can also absorb NIR light [34].

## 3. Materials and Methods

The following section details the experimental setup and the procedures used for our method and the materials used in the investigation. Low-cost electronic devices were employed in this study, and the methodology focuses on the application of the system as a medical device that records measurements. The objective of this section is to provide a clear and precise explanation of the methodology and materials used in the study.

The construction of the non-invasive glucose measurement device adheres to several medical norms and regulations to ensure safety, efficacy, and quality:

- US Food and Drug Administration (FDA) regulations: The FDA has established specific guidelines for blood glucose measurement devices, including non-invasive devices, covering aspects such as accuracy and reliability of measurements, device safety, and clarity of user instructions [28].
- International Organization for Standardization (ISO) standards: ISO 15197 [35] specifies requirements for blood glucose measurement systems for self-testing in the management of diabetes mellitus [30].
- International Electrotechnical Commission (IEC) guidelines: IEC 60601-1 [36] covers the general requirements for the basic safety and essential performance characteristics of electrical medical devices [27].

### 3.1. Design of the NIR Sensor

**Figure 1:**
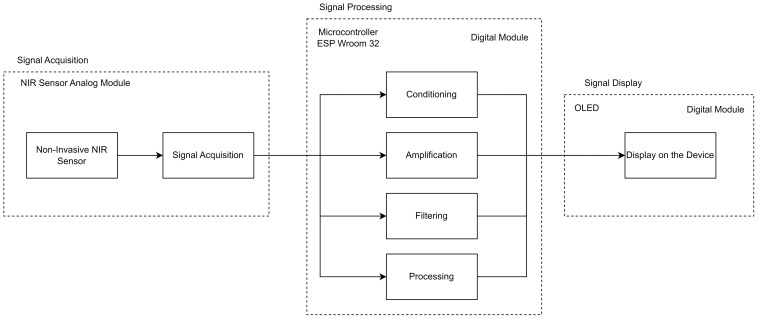
Block diagram of the instrumentation channel for device construction.

After extensive research and analysis of various technologies, near-infrared spectroscopy (NIR) was identified as the most suitable and effective method for developing a non-invasive glucose monitoring device. NIR offers several key advantages, including the ability to measure glucose non-invasively through the skin, eliminating the need for painful and frequent punctures. Moreover, NIR enables fast and accurate glucose measurements, which is critical for continuous and reliable monitoring of blood glucose levels. In the development of the prototype, an instrumentation channel was used that implements the following stages: NIR system, signal acquisition, processing (conditioning, amplification, filtering, processing, and storage), transmission, and visualization (Figure 1).

The NIR system for non-invasive glucose measurement uses near-infrared (NIR) spectroscopy to estimate blood glucose concentration from the absorption of near-infrared light by glucose in interstitial tissue. The system consists of several subsystems that work together to acquire, process, transmit, and display the NIR signal [29].

#### 3.1.1. Signal Acquisition Stage

NIR signal acquisition involves capturing near-infrared light that has interacted with the patient’s interstitial tissue. The signal acquisition subsystem consists of the following components:

- NIR light source: emits near-infrared light with specific wavelengths, typically in the range of 700 nm to 900 nm;
- NIR probe: placed in contact with the patient’s skin or mucosa, it guides the NIR light into the interstitial tissue and collects the transmitted or reflected near-infrared light;
- NIR detector: converts near-infrared light into an electrical signal.

The analog module is crucial for accurately capturing the NIR signal, amplifying it, and filtering out noise before passing it to the digital module. This module consists of two primary sub-modules: the illumination module and the capture and conditioning module.

The illumination module includes an emitting LED that emits near-infrared light at a wavelength of 940 nm. This light penetrates the finger, interacts with the interstitial tissue, and reaches the photodiode in the capture and conditioning module. The photodiode—specifically, a highly sensitive silicon-based NIR detector with a spectral range of 800 nm to 1100 nm—collects the transmitted or reflected light, converting it into an electrical signal. The detector’s high sensitivity and fast response time ensure that even minimal changes in light absorption, indicative of glucose concentration variations, are accurately captured.

The output light level of the LEDs is adjusted to a light intensity suitable for the glucose absorption rate in an electronic circuit. Four light absorption paths allow the detection of D (+)-glucose concentrations from 0 to 20% by weight in steps of 5% by weight. The light absorption process of glucose is calculated as a function of the rising edge of the PD waveform under a low-intensity light source using a time series analysis method [37].

**Figure 2:**
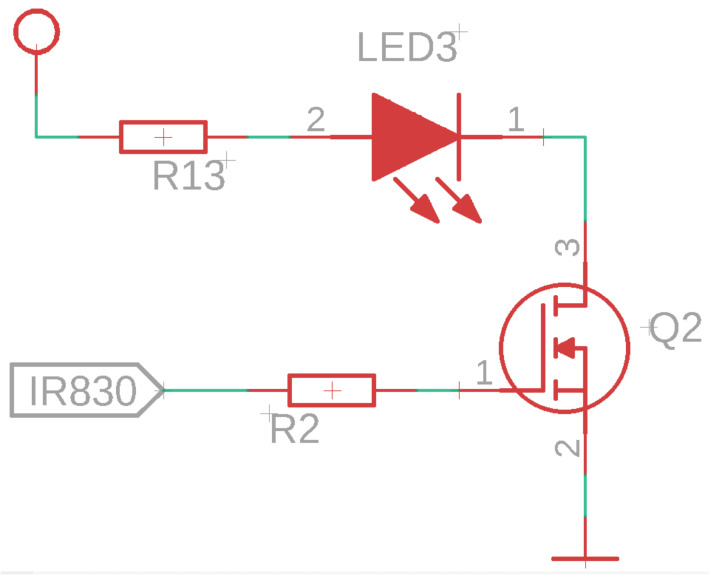
NIR sensor for glucose measurement.

The time series analysis method can be used to obtain the glucose level in PBS and reduce background noise. The application of this method improves the sensitivity of the sensor, increases the accuracy of data analysis, and reduces the cost of the measurement equipment [38]. Therefore, a two-NIR-photodiode array was implemented, as shown in Figure 2.

To maintain constant light intensity emitted by the LED1 and LED2 diodes and prevent misinterpretations during the measurement, a control circuit is implemented. This control circuit uses a MOSFET transistor to keep the current constant in each diode. The MOSFETs have a low threshold voltage with a high current, and 10 K ohm resistors are placed at their outputs to ensure proper operation.

In the capture and conditioning module, the initial signal from the photodiode is typically weak and prone to noise. Therefore, this module incorporates several stages to ensure the signal’s quality and strength. First, signal conditioning employs techniques such as shielding and grounding to minimize electromagnetic interference, thereby enhancing the clarity of the data. Then, amplification is performed using low-noise operational amplifiers to increase the signal’s amplitude, making it suitable for further processing. A transimpedance circuit is an amplifier that converts current to voltage. It is an operational amplifier (op-amp) configured with a feedback resistor in a closed loop between its output and its inverting input. The transimpedance circuit takes an input current and generates an output voltage proportional to that current, with a value of 1.6 V for sensors and 3.3 V for the controller. The relationship between the input current and the output voltage is determined by the feedback resistor. If the input current changes, the op-amp adjusts the output voltage to maintain the voltage difference between the op-amp inputs (i.e., the inverting and non-inverting inputs) close to zero.

**Figure 3:**
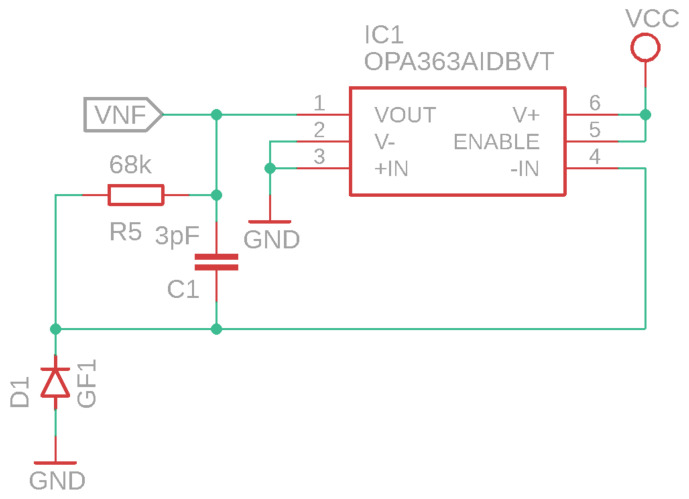
Transimpedance circuit design.

Transimpedance circuits are commonly used in applications where current needs to be converted to voltage (see Figure 3). In the case of an acquisition circuit with IR diodes, the IR diode acts as a photodiode. When IR light strikes the diode, it generates a current proportional to the intensity of the light. This current can be very small, so a transimpedance circuit is used to convert this current into a voltage that can be more easily measured. The IR diode is connected to the inverting input of the op-amp in the transimpedance circuit. The current generated by the IR diode flows through the feedback resistor, generating an output voltage in the op-amp. This voltage can then be measured and used to determine the intensity of the IR light incident on the diode.

Additionally, the analog module includes temperature compensation circuits to account for variations in ambient temperature that could affect the performance of the NIR detector. This ensures consistent signal quality regardless of external conditions. Finally, the conditioned and amplified signal is passed to the digital module for further processing and analysis.

#### 3.1.2. Signal Processing Stage

The electrical signal acquired by the NIR detector needs to be processed before the blood glucose concentration can be estimated. The signal processing subsystem consists of the following steps:

- Signal conditioning: removes noise and interference from the electrical signal;
- Amplification: increases the amplitude of the electrical signal so that it can be properly processed;
- Filtering: removes unwanted frequency components from the electrical signal;
- Processing: applies mathematical algorithms to extract relevant information from the electrical signal, such as near-infrared light absorption at specific wavelengths;
- Storage: stores the processed signal for later use.

Signal processing digitizes the signal, processes it, and sends it to the terminal device: in this case, a mobile device. Once the signal is captured and processed by the analog module, it is used by the ESP Wroom 32 board (Espressif Systems, Shanghai, China). This board is a low-cost, low-power microcontroller series with integrated Wi-Fi and Bluetooth capabilities and was developed by Espressif Systems. It has a dual-core processor that can run at up to 240 MHz, making it very powerful for a microcontroller. It supports a wide range of communication protocols, including SPI, I2C, UART, CAN, and Ethernet. It can be programmed in several languages, including C, C++, Python (through MicroPython), and JavaScript (through Espruino), making it accessible to a wide range of developers.

**Figure 4:**
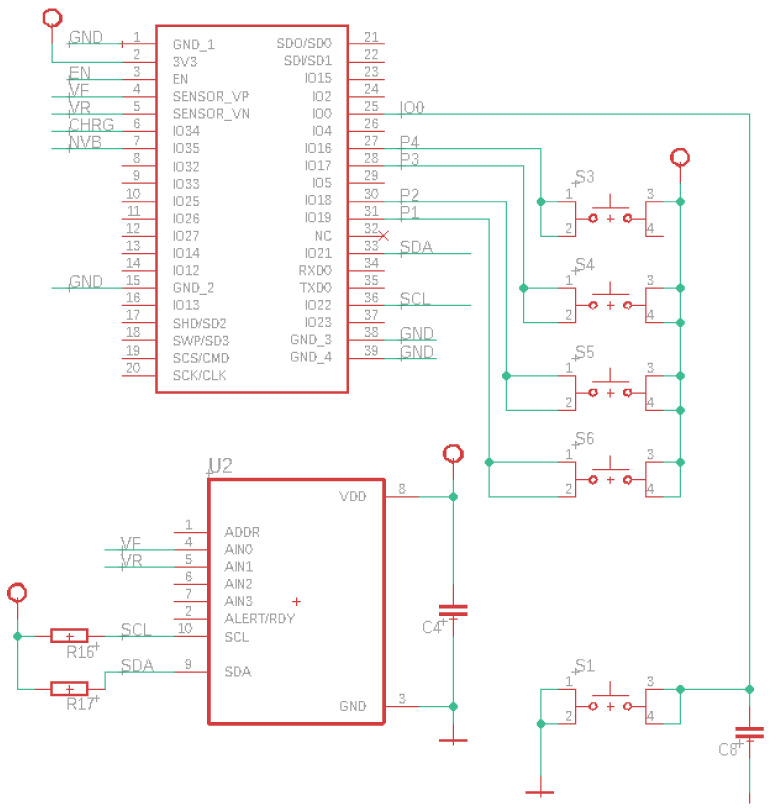
Control system.

As shown in Figure 4, the control system uses an external analog-to-digital converter (ADC) to identify subtle changes in the amplitude of the pulsatile components of the PPG signals, which are in the millivolt range. The ADC selected is the ADS1115, which has a 16-bit resolution and supports I2C communication in addition to being a low-power and low-amperage device. All communication within the device is connected via the I2C protocol. A microSD card reader is also included to store measurement data when the device lacks an internet connection.

The algorithm leverages the built-in analog-to-digital converter (ADC) of the ESP32 microcontroller, which is a powerful and versatile platform known for its low power consumption and integrated wireless capabilities. The ADC plays a pivotal role in converting the analog signals from the NIR sensors into digital values that can be processed and analyzed by the microcontroller. At the outset, the algorithm meticulously configures the ADC to optimize its performance for the specific requirements of the application. The ADC resolution is set to 12 bits, allowing for a fine-grained representation of analog values with 4096 discrete digital levels. Furthermore, the input attenuation for the relevant GPIO channels (36 and 39) is adjusted to accommodate the expected signal levels from the sensors, ensuring accurate and reliable measurements.

Notably, the algorithm operates in the ADC’s differential mode, which is a configuration that enables the measurement of voltage differences between two inputs rather than the absolute voltage of a single input. This approach enhances the precision and robustness of the measurements, mitigating potential sources of interference or noise that could compromise the accuracy of the glucose estimation process.

The core functionality of the algorithm is encapsulated within an infinite loop that is continuously reading and processing sensor data in real-time. During each iteration, the algorithm initiates the data acquisition process by invoking dedicated functions to read samples from the IR830 and IR940 sensors. These functions are designed to acquire 1000 samples for each sensor, with a brief delay of 10 ms between consecutive samples, ensuring adequate temporal resolution and minimizing potential interference from external sources.

Subsequent to the data acquisition phase, the algorithm calculates the average of the two sensor readings, effectively combining the information from both wavelengths to generate a composite representation of the glucose concentration. This average value is stored in a dedicated variable, providing a robust and reliable estimate that accounts for potential variations or anomalies in individual sensor readings.

To ensure consistent and stable operation, the algorithm introduces a delay of 100 ms before commencing the next iteration of the loop. This deliberate pause not only provides a buffer for the microcontroller to process the acquired data but also contributes to minimizing power consumption, which is a critical consideration in portable or wearable devices with limited battery capacity.

While this algorithm represents a simplified representation of the signal processing stage, it serves as a foundation for more advanced techniques and algorithms employed in state-of-the-art non-invasive glucose monitoring devices. In real-world applications, additional signal processing methodologies, calibration procedures, and complex algorithms would likely be employed to further enhance the accuracy and reliability of the glucose measurements by accounting for factors such as individual physiological variations, environmental conditions, and potential interferents. Algorithm 1 follows:
**Algorithm 1** Sensor Reading Function 1: **procedure** SensorReading 2:    **Configure ADC** 3:    Set ADC resolution to 12 bits 4:    Set channel 0 (GPIO 36) attenuation to 11 dB 5:    Set channel 3 (GPIO 39) attenuation to 11 dB 6:    Enable ADC differential mode 7:    **while** true **do** 8:      d_ir830← Read glucose using IR830 sensor with 1000 samples and 10 ms delay 9:      d_ir940← Read glucose using IR940 sensor with 1000 samples and 10 ms delay10:      glucose←(d_ir830+d_ir940)/211:      Delay for 100 ms12:    **end while**13: **end procedure**

##### Voltage Lifting and Regulation

**Figure 5:**
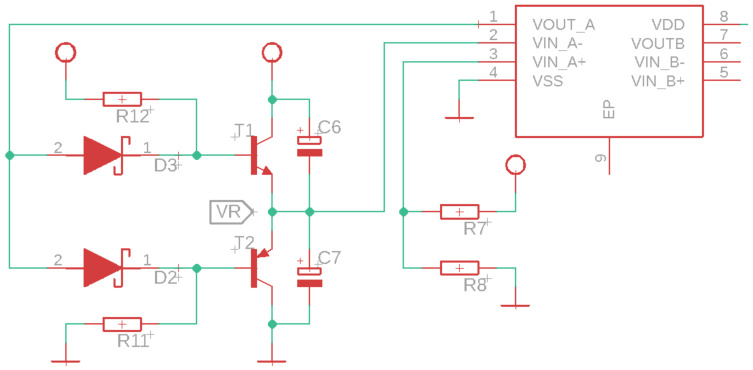
Elevation and regulation circuit.

The lifting stage is based on the voltage response of lithium-ion batteries. The voltage of these batteries decreases exponentially below 3.7 V, and most circuits using 5 V as a power supply will not function properly. Therefore, the battery voltage is raised to 7 V and is subsequently regulated to maintain a constant 5 V supply. The proposed elevation and regulation circuit is shown in Figure 5.

Most lithium-ion batteries have protection circuits to prevent the voltage from falling below or exceeding their maximum operating value. Another protection circuit was created to separate the circuits that consume energy from the battery during the charging process. If this separation is not maintained, the battery will not be fully charged, deteriorating its useful life. To achieve this, the SSM3J351R P-channel MOSFET (Toshiba, Tokyo, Japan) and the PMEG2010EJ Schottky diode (Nexperia, Nijmegen, The Netherlands) are used. During battery charging, the input voltage VDC goes to the voltage VC (with a small voltage drop of 0.3 V due to the threshold voltage of the diode), allowing the circuit to function normally while the battery charges. When the battery is not charging, the MOSFET is deactivated, and the voltage VV passes to the voltage VC so that the other circuits use the battery voltage.

#### 3.1.3. Signal Display

The algorithm responsible for reading and processing signals from the near-infrared (NIR) sensors is an integral part of the non-invasive glucose monitoring device. However, to provide a comprehensive user experience, the device incorporates an OLED (organic light-emitting diode) display that serves as a user interface, presenting the measured glucose levels and other relevant information to the end-user.

Once the sensor data have been acquired and processed by the algorithm, the resulting glucose concentration estimate is prepared for display on the OLED screen. The microcontroller, in this case, the ESP32, utilizes its built-in I2C communication protocols to transmit the processed data to the OLED display module, enabling efficient and reliable data transfer.

Upon receiving the processed data from the microcontroller, the OLED display module renders the information in a user-friendly format. This may include displaying the current glucose level as a numerical value, accompanied by units of measurement (e.g., mg/dL or mmol/L).

Furthermore, the device incorporates an alarm system that alerts the user in case of critical situations, such as dangerously high or low glucose levels. When the processed data indicate that the glucose concentration has exceeded predefined thresholds, the microcontroller triggers an alarm signal that is transmitted to the OLED display. In response, the display may present a visual warning, such as flashing icons or color-coded indicators, accompanied by audible or vibration alerts depending on the device’s capabilities.

By seamlessly integrating the sensor data processing algorithm with the OLED display and alarm system, the non-invasive glucose monitoring device offers a comprehensive solution that not only accurately measures glucose levels but also presents the information in a user-friendly manner, empowering individuals to proactively manage their health and well-being.

### 3.2. Circuit Implementation

The construction of a non-invasive glucose measuring device requires strict compliance with medical standards and regulations to ensure its safety, effectiveness, and quality. Key guidelines include those from the US Food and Drug Administration (FDA), which set specific standards for measurement accuracy, reliability, device safety, and user instructions. Additionally, the International Organization for Standardization (ISO) established several relevant standards: notably, ISO 15197, which specifies requirements for blood glucose measurement systems for self-monitoring in diabetes management.

Furthermore, the International Electrotechnical Commission (IEC) provided essential guidelines for the safety of electrical medical devices, with IEC 60601-1 covering general requirements for basic safety and essential performance. Adhering to these standards is crucial when developing a reliable, accurate, and user-friendly non-invasive glucose monitoring device that meets regulatory expectations and ensures patient safety.

**Figure 6:**
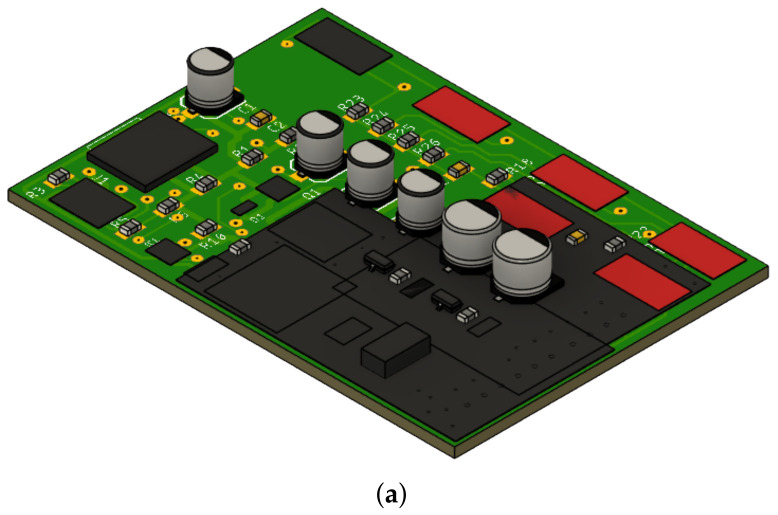

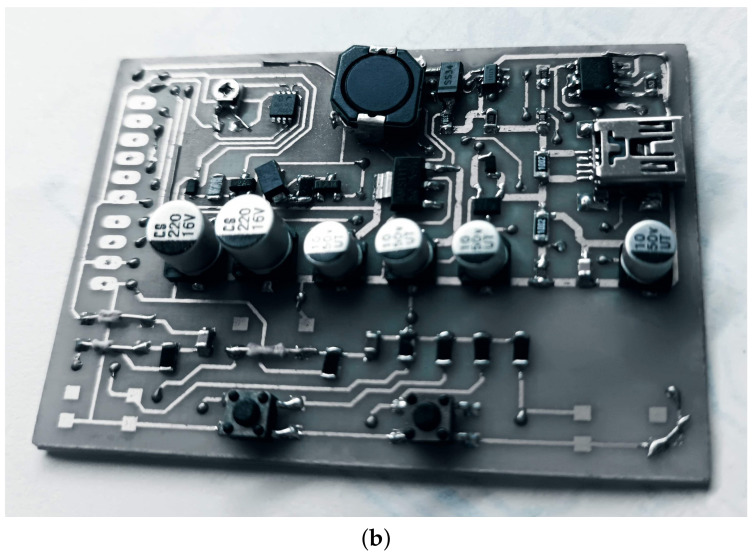
Circuit implementation: (**a**) Layout design of the control board created using Eagle software. (**b**) Real PCB of the control board, manufactured based on the layout design generated using Eagle software.

Figure 6a displays the control board, which was meticulously crafted using Eagle software v. 9.6.2 which is a renowned PCB design tool developed by Autodesk (San Francisco, CA, USA). The acronym ’Eagle’ stands for ’Easily Applicable Graphical Layout Editor’, reflecting its user-friendly interface and powerful capabilities. Widely embraced by electronic engineers and PCB designers, Eagle facilitates seamless schematic creation and PCB layout design. Leveraging Eagle’s extensive library of electronic components streamlines the selection and placement process, ensuring precise integration of components onto the board. Moreover, Eagle’s comprehensive design validation features enable users to meticulously inspect designs for electrical and layout errors before moving on to manufacturing. This proactive approach helps preemptively address potential issues, minimizing the risk of costly errors during the fabrication process [27].

Additionally, in Figure 6b, alongside the rendered layout design, are the real-life PCBs of the circuit, which was meticulously crafted based on the Eagle design. This juxtaposition offers a visual representation of the translation from digital design to physical implementation, showcasing the accuracy and fidelity achieved in replicating the layout onto the physical board. The integration of both the digital design and its real-world counterpart provides a holistic view of the development process, highlighting the precision and attention to detail required in electronic engineering endeavors.

In parallel, Fusion 360 (v.16.7.0.2155) was utilized for the design; Fusion 360 is CAD software that was also developed by Autodesk. Fusion 360 is a comprehensive tool that combines industrial and mechanical design, simulation, collaboration, and machining. It enables users to design and model products in 3D, perform analyses and simulations to test design functionality, and generate machine code for manufacturing prototypes and final products. Its cloud collaboration capabilities facilitate teamwork and project management [39].

**Figure 7:**
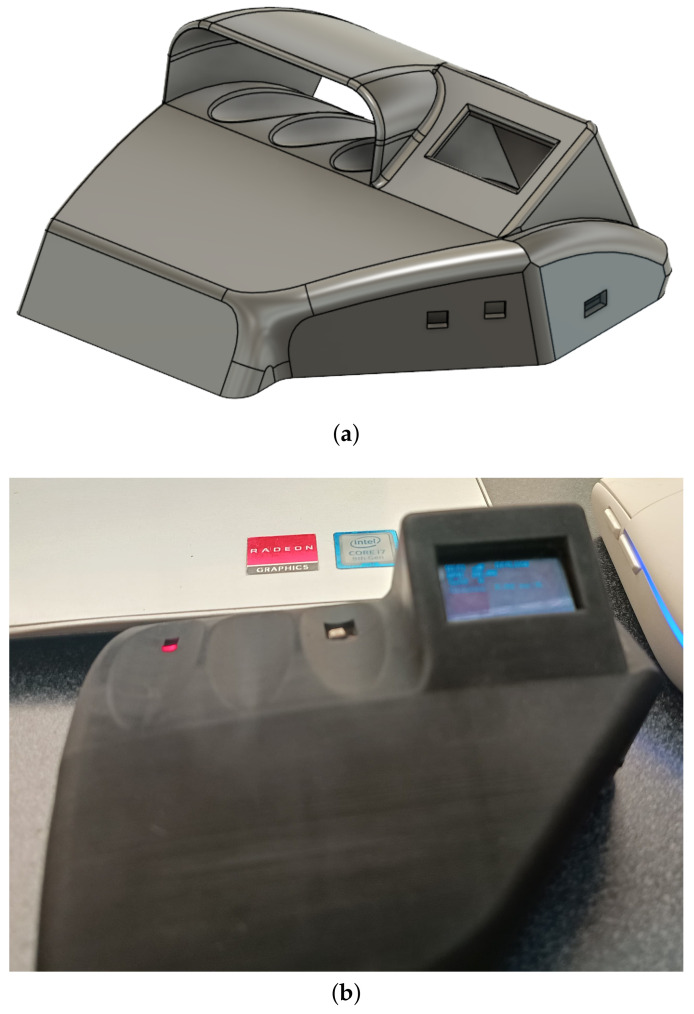
Ergonomic case: (**a**) Design preview of the electronic control board and protective case, which were created using Fusion 360 CAD software. (**b**) Manufactured electronic control board and protective case, showcasing the final product after 3D printing.

Using Fusion 360, we obtained a preview of the design in 3D, as illustrated in Figure 7a, which aided with designing the structure that supports the control board. The protective case for the control board was designed to be ergonomic, easy to handle, and intuitive to use. The sensors were positioned to facilitate easy reading and comfort; we selected the ring and index fingers for optimal placement. Additionally, the preview designed in Fusion 360 was manufactured using a 3D printer (see Figure 7b), which provided a physical representation of the digital model. This allowed for practical testing and validation of the design’s functionality and ergonomics before finalizing the production of the protective case.

## 4. Results

In this section, we present the outcomes of our investigation into the effectiveness and reliability of the non-invasive glucose monitoring device. Through rigorous testing and analysis, we aim to evaluate the device’s performance at accurately measuring blood glucose levels and its potential impact on diabetes management. The results offer valuable insights into the device’s functionality, highlighting its strengths and areas for improvement. By examining various aspects such as accuracy, stability, and ease of use, we contribute to the ongoing discourse on non-invasive glucose monitoring technology, ultimately striving to advance the field and enhance patient care.

### 4.1. Functionality Test

Extensive testing was conducted on an electronic biosignal monitoring system that was designed for the prevention and management of diabetes mellitus. This system utilizes near-infrared spectroscopy to accurately measure blood glucose levels. Compared to various commercial devices, this system exhibited outstanding performance, delivering precise results with an error margin of less than 3%. Testing involved a diverse group of 120 individuals spanning different ages, genders, and stages of diabetes in order to evaluate the system’s efficacy in real-world scenarios. The results confirmed the system’s ability to consistently and accurately measure blood glucose levels across a wide demographic range.

To assess the system’s reliability, relative error calculations were performed for each photodiode case based on its admittance, revealing an error rate of less than 5%. This aligns with medical standards, indicating successful data collection and compliance with measurement protocols. Table 1 illustrates the system’s reliability percentage through a comparison with a commercial glucometer, showcasing the relative error as a percentage measure of deviation from reference values. This metric provides insight into the magnitude of error relative to the actual value, aiding with gauging measurement accuracy.

The non-invasive glucose monitoring device utilizes near-infrared (NIR) photodiode technology to provide accurate and continuous measurements of blood glucose levels. NIR photodiodes function by emitting light in the near-infrared spectrum towards the patient’s skin. This light interacts with molecules in the skin, including glucose, which absorbs some of the light.

The measurement process commences when the NIR photodiodes emit light towards the patient’s skin. Some light photons are absorbed by glucose and other molecules present, while the remaining light is reflected and collected by the photodiodes. This reflection generates an electrical signal containing information about the absorbed and reflected light.

To ensure accurate glucose measurement, the electrical signal collected by the NIR photodiodes undergoes a filtering process. This process eliminates interference and noise, such as variations in the skin due to blood circulation or the presence of other molecules that might affect the measurement.

Once the signal is filtered, the device analyzes the patterns and characteristics of the light absorbed by glucose. This analysis is performed using algorithms and signal processing techniques that identify and quantify the glucose concentration in the sample.

Finally, the device computes the blood glucose level from the processed data. This measurement is displayed on a visual interface such as an OLED screen, allowing patients and medical staff to view the results clearly and accurately. Additionally, the measurement data are stored in a database for further access and analysis, enabling long-term tracking of the patient’s metabolic status.

### 4.2. Analysis and Discussion of Results

To analyze the collected data, various statistical techniques were employed to evaluate the effectiveness of the biosignal monitoring system. This analysis involved T-tests to compare group means and analysis of variance (ANOVA) to examine interactions between variables.

Absorbance, measured using the Beer–Lambert law, was utilized to assess the quality of light absorbed by the sample. The Beer–Lambert law states that the absorbance of a sample is proportional to its concentration and the path length of light through the sample:

(1) $$A=log_{10}\left(\frac{I_{0}}{I}\right)$$

**Figure 8:**
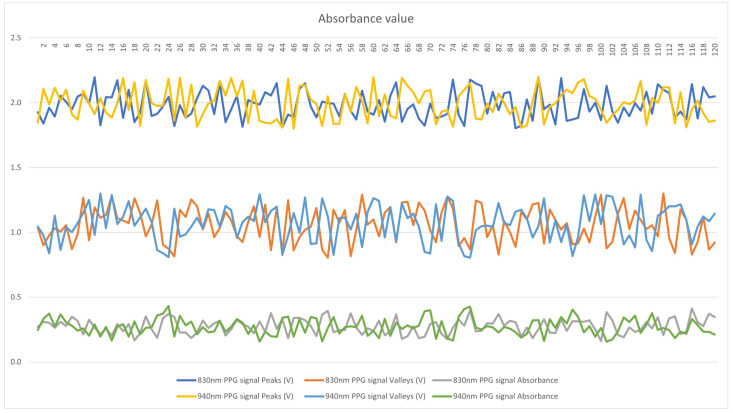
Absorbance values.

In Equation (1), absorbance is the base 10 logarithm of the ratio between the intensity of incident light ($I_{0}$) and the intensity of transmitted light (*I*). It is essential to note that the Beer–Lambert law assumes the sample is homogeneously mixed and that light absorption is uniform. Deviations from these conditions may result in inaccurate outcomes. Figure 8 presents the absorbance values derived from the PPG signals for each IR photodiode.

The study examined differences in blood glucose levels between the control and experimental groups. A correlation analysis was conducted to explore the relationship between biosignal monitoring system usage and diabetes control variables. The results can be seen in Figure 8:

Figure 8 displays absorbance values calculated from the infrared spectrum using the PPG values, which aid with quantifying the substance concentration in a sample. The amount of light absorbed correlates directly with the substance’s concentration.

Tests and evaluations revealed a significant correlation between the measurements obtained by the device and the blood glucose values from conventional invasive methods such as glucometers. The key highlights include the accuracy and stability of the device’s measurements, which demonstrate high agreement with standard methods. This reliability is crucial for clinical validation and acceptance of the device for real-time glucose monitoring.

Additionally, the speed and efficiency of the measurement process were assessed. The device’s rapid results acquisition makes it suitable for continuous and frequent glucose monitoring, which is essential in diabetes management for timely clinical decision-making.

The ease of use and patient comfort were also evaluated. The device’s non-invasive nature and straightforward operation enhance user accessibility and convenience, potentially improving adherence to regular monitoring. This is vital for effective diabetes management and complication prevention. Although promising results were obtained, further research and refinement are necessary to optimize system performance and accuracy. Consideration should also be given to integrating additional functions, such as connectivity with mobile devices and alerts for significant glucose level variations.

Near-infrared (NIR) glucometry offers several advantages over traditional finger-prick methods, being painless and non-invasive and facilitating frequent blood glucose monitoring. This can improve glycemic control and patient quality of life. However, challenges remain before widespread adoption, including enhancing NIR device accuracy for clinical reliability and conducting broader studies to evaluate long-term effectiveness in glycemic control and diabetes complication prevention.

**Figure 9:**
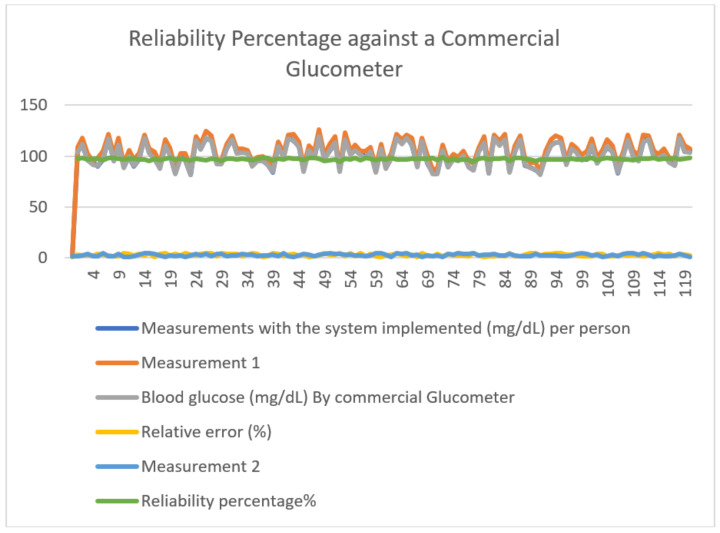
Reliability Percentage against a Commercial Glucometer.

Reliability calculations showed that both the commercial and research devices have a similar reliability margin, as depicted in Figure 9, with an average accuracy value of 97.117% compared to a commercial glucometer.

The results demonstrate the biosignal monitoring system’s effectiveness in diabetes prevention and control, highlighting its potential to revolutionize diabetes management. Future research and device design improvements that are tailored to patient and specialist needs could integrate this technology into existing diabetes care systems.

## 5. Discussion

The findings of this study align with previous research demonstrating the feasibility and accuracy of near-infrared (NIR) technology in non-invasive glucose measurement. The high level of agreement observed between the device measurements and the blood glucose values obtained by invasive methods supports the reliability and utility of the non-invasive monitoring system. These results corroborate previous studies highlighting NIR technology’s ability to provide accurate and rapid glucose measurements, making it a promising tool for continuous diabetes monitoring.

Patient comfort and ease of use are critical aspects addressed in this study and have been emphasized in prior research. The elimination of punctures and the simplicity of device operation may enhance adherence to monitoring, which is essential for effective long-term diabetes management. The results obtained in this study, when compared with the existing literature, affirm the effectiveness and relevance of the non-invasive glucose monitoring device based on NIR photodiodes. These findings contribute to technological advancements in diabetes and glucose monitoring, opening new possibilities for personalized and effective management of this chronic disease.

Despite the promising results achieved with the near-infrared spectroscopy technique for non-invasive glucose measurement, there is potential to further increase precision by combining this technique with other non-invasive methods, such as impedance spectroscopy. Integrating both techniques could reduce the margin of error and yield more accurate and reliable measurements.

Various factors such as movement during measurements, temperature fluctuations, and pressure changes in the sensor can affect data collection. Therefore, it is advisable to use compensation methods that significantly reduce these errors. Techniques such as electrical impedance analysis, sweat measurement, light transmission through fabrics, and photoacoustics could be employed to mitigate these external influences.

Developing an electronic biosignal monitoring system for diabetes mellitus prevention and control based on spectroscopy is a significant yet promising technological challenge. Extensive validation, including compensation for external factors and clinical studies, is recommended to evaluate its accuracy and efficacy compared to invasive methods. Exploring continuous monitoring capabilities and connectivity options could further enhance patient monitoring and management.

This section provides a concise and precise description of the experimental results, their interpretation, and the conclusions drawn, thereby contributing to the advancement of non-invasive glucose monitoring technology.

## 6. Conclusions

The evaluation of electronic biosignal monitoring systems is fundamental for determining their suitability and effectiveness in medical and preventive applications. Near-infrared (NIR) spectroscopy stands out as one of the most effective methods for detecting and controlling diabetes mellitus. This method demonstrated high precision and accuracy for glucose measurements compared to traditional approaches. Although a relative absorbance error margin of around 3% was observed, this value remains highly reliable for monitoring diabetes patients.

Using NIR spectroscopy in glucose measurement aligns with the guidelines established by the American Diabetes Association (ADA). By implementing NIR spectroscopy, progress has been made towards less invasive and more comfortable data collection for diabetes patients. Regular monitoring of glucose levels is essential for maintaining them within healthy ranges, which reduces long-term complications related to the disease. For type 1 diabetes, meticulous control of glucose values is vital due to the reliance on insulin administration. For those with type 2 diabetes, maintaining appropriate glucose levels helps avoid insulin resistance and improve sensitivity. This reduces chronic complications and allows for an active life. Education on the importance of monitoring, involvement of healthcare professionals, and the use of reliable technologies improve the lives of people living with diabetes.

The design of an electronic biosignal monitoring system using NIR spectroscopy with the strategic incorporation of two photodiodes at wavelengths of 820 and 960 nanometers represents a promising advancement in blood glucose monitoring for people with diabetes. NIR spectroscopy allows for the indirect estimation of glucose levels, eliminating the need for painful punctures. The selection of wavelengths is based on the differential absorption of glucose and other blood components, allowing for accurate estimations of glucose levels when properly processed. This non-invasive design reduces the physical and emotional discomfort associated with traditional punctures, facilitating regular monitoring of glucose levels and enabling patients to make informed decisions about their treatment, diet, and lifestyle.

During the sample collection process, a relative absorbance error of approximately 3.2% was observed in the 820 and 960 nanometer photodiodes. While this error margin is small, it is crucial to consider this variability to ensure the accuracy and reliability of the electronic biosignal monitoring system. An exhaustive analysis must be conducted to identify the source of this error and implement corrective measures to minimize its impact on future measurements. Despite this challenge, the system demonstrated remarkable effectiveness overall. NIR spectroscopy has proven to be a promising technology, and while challenges remain, such as addressing the margin of error in the 820 and 960 nanometer photodiodes, the evaluation results strongly support the adoption and ongoing development of electronic biosignal monitoring systems based on this technique for the control and prevention of diabetes mellitus.

The use of 240 samples in the study provides a solid database to support the results obtained with the electronic monitoring system. This representative sample ensures the robustness and validity of the findings, contributing to the credibility of the device and its potential adoption in clinical settings. According to the data obtained in Table 1, the comparison with values from a traditional glucometer shows that the electronic biosignal monitoring system significantly outperforms traditional methods. This suggests that the use of NIR spectroscopy can successfully replace conventional monitoring methods, improving the accuracy and quality of care for diabetic patients. The application of NIR spectroscopy in the electronic biosignal monitoring system for the prevention and control of diabetes mellitus has proven to be highly effective, with an accuracy rate of 97%. This supports the suitability and reliability of the technique for detecting and monitoring glucose levels in patients with type 1 and type 2 diabetes.

Future work should focus on enhancing the accuracy and robustness of the electronic biosignal monitoring system. Integrating additional non-invasive measurement techniques such as impedance spectroscopy could help reduce the margin of error and improve overall reliability. Advanced compensation methods to address external factors such as movement, temperature, and pressure changes are also crucial. Further clinical trials involving larger and more diverse patient populations are necessary to validate the system’s effectiveness across different demographics and conditions. Exploring connectivity options, such as integration with mobile devices and cloud-based platforms, can facilitate real-time monitoring and data analysis and offer timely alerts for significant glucose variations. Additionally, investigating the long-term impact of continuous NIR monitoring on diabetes management and patient outcomes will provide valuable insights into its practical applications and benefits.

## Figures and Tables

**Table 1:** Percentage of reliability of the system according to the comparison with a commercial glucometer.

Measurements	Measurements with the Implemented System		Blood Glucose Commercial	Relative Error (%)		Reliability Percentage
	**1**	**2**	**Glucometer**	**1**	**2**	**%**
1	106	109	104	3.150	1.810	97.604
2	111	118	112	2.390	2.250	97.919
3	96	102	96	2.290	4.020	96.714
4	94	95	92	2.450	1.690	97.738
5	90	98	91	3.530	1.330	97.330
6	97	105	98	3.450	4.800	95.791
7	118	122	117	2.750	1.950	97.991
8	95	101	95	1.510	1.310	98.516
9	110	118	111	1.440	4.230	97.446
10	91	92	89	4.830	1.220	96.582

## Data Availability

No new data were created or analyzed in this study. Data sharing is not applicable to this article.
